# The Effect of Ionising Radiation on the Properties of Tumour-Derived Exosomes and Their Ability to Modify the Biology of Non-Irradiated Breast Cancer Cells—An In Vitro Study

**DOI:** 10.3390/ijms26010376

**Published:** 2025-01-04

**Authors:** Michał Stefan Lach, Joanna Patrycja Wróblewska, Marcin Michalak, Bartłomiej Budny, Elżbieta Wrotkowska, Wiktoria Maria Suchorska

**Affiliations:** 1 Department of Electroradiology, Poznan University of Medical Sciences, Garbary 15, 61-866 Poznan, Poland; wiktoria.suchorska@wco.pl; 2 Radiobiology Lab, The Greater Poland Cancer Centre, Garbary 15 Street, 61-866 Poznan, Poland; 3 Department of Life Sciences and Medicine (DLSM), University of Luxembourg, 6, Avenue du Swing, 4367 Belvaux, Luxembourg; joanna.wroblewska@uni.lu; 4 Surgical, Oncological and Endoscopic Gynaecology Department, The Greater Poland Cancer Centre, Garbary 15 Street, 61-866 Poznan, Poland; marcin.michalak@wco.pl; 5 Department of Endocrinology, Metabolism and Internal Diseases, Poznan University of Medical Sciences, Przybyszewskiego 49 Street, 60-355 Poznan, Poland; bbudny@ump.edu.pl (B.B.); ewrotkowska@ump.edu.pl (E.W.)

**Keywords:** exosomes, breast cancer, radiotherapy, angiogenesis, ionising radiation

## Abstract

The vast majority of breast cancer patients require radiotherapy but some of them will develop local recurrences and potentially metastases in the future. Recent data show that exosomal cargo is essential in these processes. Thus, we investigated the influence of ionising radiation on exosome properties and their ability to modify the sensitivity and biology of non-irradiated cells. Exosomes were isolated from breast cancer cell lines (MDA-MB-231, MCF7, and SKBR3) irradiated with 2 Gy (Exo 2 Gy) or no irradiation (Exo 0 Gy). Despite some differences in their molecular profiles, they did not affect cell viability, proliferation, cell cycle phase distribution, and radioresistance; however, both populations showed the ability to modify cell migration and invasion potential, as confirmed by the downregulation of β-catenin, which is responsible for maintaining the epithelial phenotype. Interestingly, exosomes from irradiated BCa cells were more actively deposited in the endothelial cells (EA.hy926). Furthermore, exosomes tend to lower the expression of CD31, which is responsible for maintaining intact vascularity. This preliminary study demonstrates the vital role of exosomes and their altered profile due to irradiation in the pathobiology of breast cancer.

## 1. Introduction

Among all cancers, breast cancer (BCa) remains a leading cause of death among women with diagnosed malignancies. Globally, by 2022, almost 2.3 million new cases were diagnosed, and approximately 670,000 patients have died [1]. With tremendous progress in developing new targeted therapies and prophylaxis programs, mortalities have declined over the last few decades [2]. The complexity of this disease has led to several approaches that rely on a combination of surgical interventions, hormone therapy, chemotherapy, radiotherapy, and targeted therapy [3]. Typically, surgical removal of the malignancy with a combination of postoperative irradiation is performed for locoregional treatment. The standard treatment regimen for over several decades included whole breast irradiation (WBI) using fractions (1.8–2.0 Gy) delivered for 5 to 7 weeks [4,5,6,7]. The main goal of radiotherapy is to eradicate highly proliferative malignant cells by damaging their deoxyribonucleic acid (DNA) with minimal risk to surrounding healthy tissue [8,9,10]. This is achieved in two ways: direct mechanisms, where energy is deposited in the nucleic acid macromolecule caused by charged particles, or indirect mechanisms, based on DNA depletion through interaction with reactive oxygen species (ROS) generated from water radiolysis [10,11]. As a consequence, double-strand breaks (DSB) and single-strand breaks (SSB) occur, which can lead to mutations, genetic instability, mitotic catastrophe, and cellular death. The relative biological effectiveness (RBE) of cells exposed to ionising radiation is dependent on a few parameters, including the linear transfer energy (LET), where for low LET (for example, X-rays, gamma rays, and electrons), the vast majority of their effect occurs on an indirect basis, whereas in the case of high-LET radiation (e.g., protons and carbon ions), direct damage is the predominant mechanism of action. Moreover, low LET causes more dispersed DSB and SSB, which are more easily repaired by cells than more complex and clustered DNA lesions caused by high-LET radiation [10,12,13]. Despite the best efforts, detailed dosimetry planning, and initial good responses, some patients experience recurrence, especially those diagnosed at advanced stages [6,14].

One possible cause of therapeutic failure could involve the presence of functional structures secreted by cells called extracellular vesicles. Among these structures, those originating from membranous intracellular compartments and within the size of virus-like particles (30–150 nm), a population called exosomes, have been extensively studied [15,16]. They have gained considerable attention in recent decades owing to their unique properties and involvement in several physiological and disease-related processes. Exosomes play a unique role in cancer biology [17,18]. They are associated with immune-escape regulation, the development of resistance to several therapeutic approaches, and the regulation of local and distant spread of cancer by remodelling the cells composing the local and distal tissue microenvironment [19,20,21,22,23,24]. Moreover, several studies suggest that tumour-derived exosomes (TEX) could be utilised as potential early diagnostic and disease progression markers in several malignancies due to their unique cargo composition [15,25,26]. Moreover, they are considered to be good predictive markers of therapeutic response [27,28,29]. That is possible because their volume, compared to the total concentration of exosomes in the bloodstream, is elevated in patients with neoplasms and correlates with the advancement of the disease [18,26,30,31].

The cargo and properties of exosomes are highly dependent on the condition of the donor cell and can be affected by chemical and physical factors. One of them, ionising radiation, is responsible for tremendous changes in the physiology of cells by direct or indirect damage/interaction with their structures and molecules [10,11]. The induction of exosomal secretion is also disturbed and partially altered by domination, one of the pathways involved in the increased expression of tumour suppressor-activated pathway 6 (TSAP6), a protein induced by p53 activation as a consequence of DNA damage response (DDR) to irradiation [32,33,34]. On top of that, the small GTPases (i.e., Ras-related proteins 11 (Rab11), Rab27a, and Rab27b) involved in the biogenesis and secretion of exosomes are also disrupted, which could be observed by a positive correlation between their expression and the irradiation dose absorbed by the cell [33,34]. Furthermore, there is growing evidence that exosomes from irradiated compartments of the organism are involved in the radiation-induced bystander effect (RIBE) and even radiation-induced abscopal effect (RIAE) [35,36,37,38,39]. They are ideal candidates for these processes because their size, structure, and composition enable them to migrate along the bloodstream and spread all over the body, including crossing the blood–brain barrier (BBB) [35,40]. The RIBE has a dualistic nature—on the one hand, it can damage cells locally and distally, leading to their dysfunction, such as radiation-induced cognitive impairment [41], injury of intestinal crypts [42], or the inhibition of wound healing [43]; on the other hand, it can stimulate malignant cells to transduce signals required for the development of radioresistance by enhancing genes responsible for efficient DNA repair and survival, promoting their proliferation, migration, and genomic instability (GI) [38,44,45,46,47,48,49,50]. However, little is known about their properties after radiotherapy and their effect on the non-exposed surrounding malignant and normal cells at clinically relevant doses used during fractionated radiotherapy. To date, there have been published data on tumours such as head and neck, glioblastoma, pancreatic, lung, prostate, and oesophageal, with only limited data regarding breast cancer [34,44,45,49,50,51,52,53,54,55,56,57,58,59]. They have provided some primary insights into the possible roles of exosomes as universal carriers of molecules responsible for the indirect radiation response in non-exposed cells. In the breast cancer cell line MCF-7, exosomal cargo from irradiated cells (proteins and non-coding RNA) can modulate the genetic instability of co-cultured cells, telomere shortening for several population doublings, and for shorter observation periods, induce increased invasiveness [44,48,49,60]. Some preliminary studies suggest that they can transfer/induce radioresistance in resistant cells or with a 4 Gy dose treatment of MDA-MB-231 cells [53,54].

In the literature, there lack of studies testing a broad panel of BCa cell lines and establishing the dualistic effects of exosomal cargo from irradiated cells to non-treated BCa cells and how their biology is affected. Moreover, there is a lack of information on how they could affect endothelial cells, which is crucial for the further spread of cancer cells. In this study, we attempted to fill this gap and add more information regarding the effect of exosomes—isolated from cells exposed to a clinically relevant irradiation dose—on a broad spectrum of properties of unirradiated cells.

## 2. Results

### 2.1. Irradiation Affected the Composition of Exosome-Related Proteins

**Figure 1:**
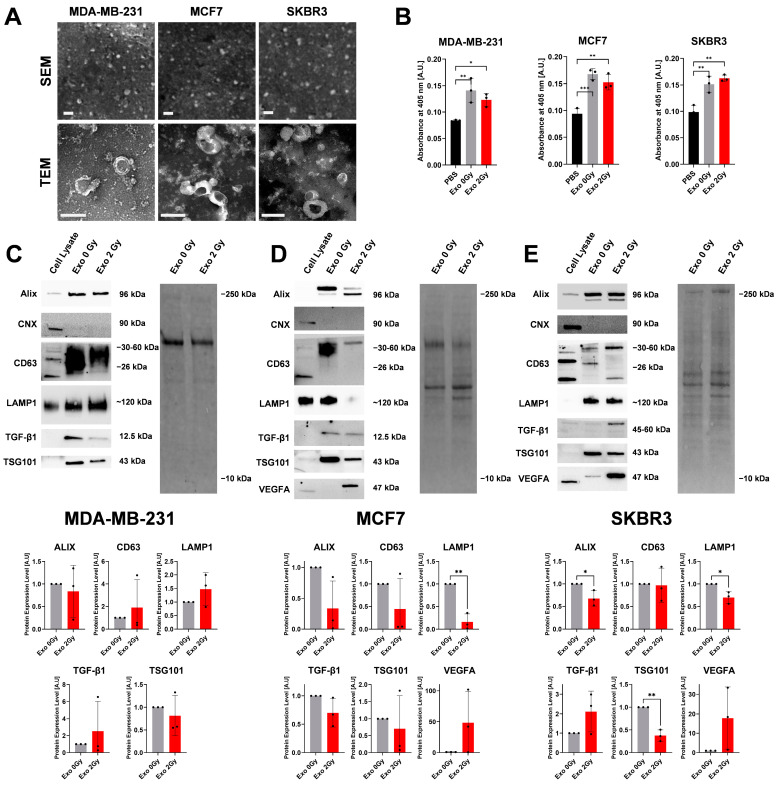
The characteristics of exosomes isolated from non- and irradiated BCa cell lines. (**A**) The representative pictures of isolated exosomes using scanning and transmission electron microscopy. The white scale bar represents 200 nm. (**B**) The acetylcholinesterase activity assay in isolated exosomes from cell culture medium. The graph bars represent mean ± SD from three independent experiments (black dots are the mean of technical triplicates). The statistical comparisons were calculated using ANOVA with Tukey’s post hoc test for multiple comparisons. *p* < 0.05 *; *p* < 0.01 **; *p* < 0.001 ***. PBS—control. (**C**–**E**) The representative blots show the successful isolation of the exosomes using Western blot techniques with assessment of changes in the expression of exosomal markers (Alix, CD63, LAMP1, TSG101) and functional cargo (TGF-β and VEGFA) due to exposure of cells to ionising radiation. On the left is the representative total protein lane used for quantification of the signal. The graphs represent the mean ± SD normalised to the intensity signal from the total protein content. The black dot represents a single replicate of independent isolation. The statistical significance was determined using an unpaired two-tailed Student’s *t*-test. *p* < 0.05 *; *p* < 0.01 **.

To confirm the successful isolation of the exosomes, we have conducted the following procedures: scanning and transmission electron microscopy and enzymatic activity of acetylcholinesterase (AChE) (Figure 1A,B). Among all the tested BCa cell lines, we observed the presence of membrane nanoparticles smaller than 200 nm, which is typical for exosome morphology and represents a cup-like shape. The AChE activity is sometimes allocated to the presence of exosomes but we are aware that it is not their typical marker. The absorbance of Exo 0 Gy and Exo 2 Gy were significantly higher than the control amid the tested MDA-MB-231, MCF-7, and SKBR3 cell lines compared to PBS; however, the irradiation did not cause an enhanced absorbance level neither within the exosomes nor in all tested BCa cell lines.

The analysis of protein expression of exosomal cargo from isolated BCa cell lines has confirmed the isolation of the pure population of exosomes (Figure 1C–E), which was determined by the presence of proteins related to ALIX, CD63, LAMP1, and TSG101 (typical markers of exosomal origin) and lack of organelle contamination (negative for the presence of calnexin (CNX)). In addition, we have tested functional proteins such as TGF-β (involved in several processes, i.e., fibrosis, senescence, and immunosuppression) and VEGFA (angiogenesis). The detailed comparison of protein expression levels in exosomes derived from MDA-MB-231 revealed no statistical differences between Exo 0 Gy and Exo 2 Gy. Moreover, we were unable to detect VEGFA in their cargo. In MCF7-derived exosomes, the ALIX, CD63, LAMP1, TGF-β, and TSG101 proteins displayed a decreasing tendency between Exo 2 Gy and Exo 0 Gy; however, only in LAMP1 we observed statistically meaningful changes. VEGFA expression in MCF7-derived exosomes showed some increase in signal intensity but did not reach statistical significance. On the other hand, in the SKBR3-derived exosomes, the ALIX, LAMP1, and TSG101 proteins did show a meaningful drop in the Exo 2 Gy variant compared to Exo 0 Gy. The CD63 exosomal levels were undistinguishable between tested groups. In the case of TGF-β and VEGFA, a statistically insignificant elevation in the protein level was notified.

### 2.2. Effect of Irradiated Exosomes on BCa Cell Properties

**Figure 2:**
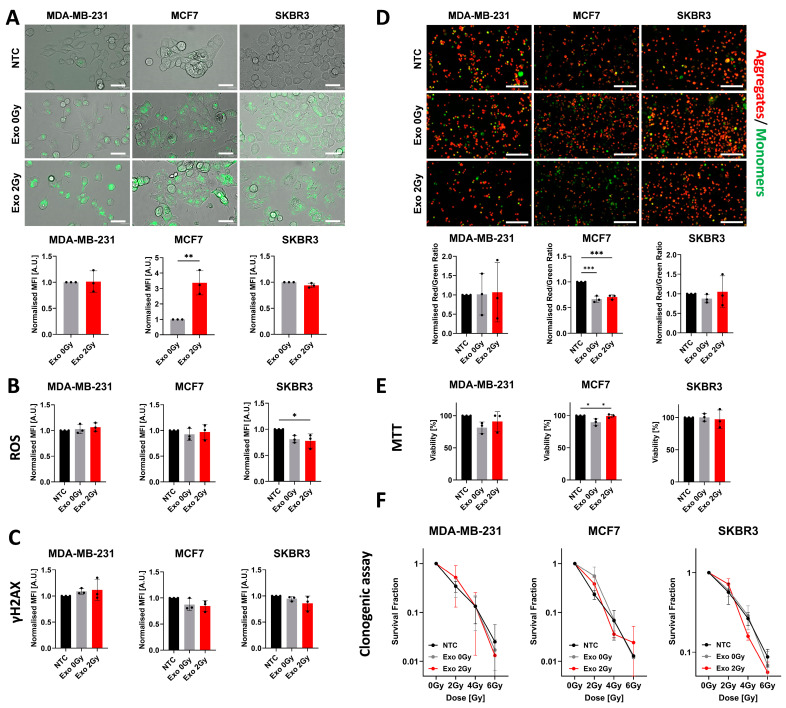
The effect of exosomes isolated from non- and irradiated BCa cell lines on the uptake, reactive oxygen species formation, γH2A.X presence, mitochondrial membrane depolarisation, proliferation, and radioresistance. (**A**) The microscopic and flow cytometric evaluations of exosome uptake by EA.hy926 cell line after 4 h. The representative picture of endothelial cells actively uptake PKH67-labelled exosomes. The white scale bar represents 40 µm; pictures were taken under 400× magnification. The graphs represent the normalised mean ± SD from three independent experiments (the black dot represents the mean of the median fluorescence intensity (MFI) from a technical duplicate). The significance was determined using an unpaired two-tailed Student’s *t*-test. *p* < 0.01 **. The flow cytometric assessment of the ROS formation (**B**) and γH2AX (**C**) in the cells exposed to exosomes from non- and irradiated cells. The graphs represent the normalised mean ± SD from three independent experiments (the black dot represents the mean MFI from a technical duplicate). The statistical significance was estimated using ANOVA with Tukey’s post hoc test for multiple comparisons. *p* < 0.05 *. (**D**) The microphotographs show the JC-1 aggregates (red) and monomers (green) determine the mitochondrial membrane condition after exposure to exosomes isolated from non- or irradiated BCa cell lines. The white scale bar represents 400 µm; pictures were taken under 40× magnification. The flow cytometric results are presented on the graph (black dot equals the mean from the duplicates of one independent experiment) as the normalised mean ± SD of red-to-green MFI ratio. The statistical significance was determined using ANOVA with Tukey’s post hoc test for multiple comparisons; *p* < 0.001 ***. (**E**) The viability/proliferation was determined using MTT. The graphs describe the mean ± SD percentage (black dot corresponds to the mean from the triplicates of one biological experiment). The statistical differences were determined using ANOVA with Tukey’s post hoc test for multiple comparisons; *p* < 0.05 *. (**F**) The survival fraction analysis of cells exposed to irradiation using clonogenic assay after co-culture with or without exosomes. The dots on the survival curve represent the mean ± SD from the triplicates of three independent experiments. There was a lack of statistically significant differences between tested variants.

Several assays have been performed to validate the effect of exosomes derived from non- and irradiated BCa cell lines to observe the potential consequences of the potential changes in their cargo (Figure 2).

Firstly, we wanted to find out how the irradiation changes the exosome’s ability to be actively uptaken by recipient cells (Figure 2A). Only in MCF7 did we observe significant increases in the MFI of cells exposed to Exo 2 Gy. In MDA-MB-231 and SKBR3, irradiation did not cause a higher uptake of exosomal cargo.

Then, we tested their effect on the production of ROS, whether this process was abrogated or enhanced by the bystander effect (Figure 2B). In MDA-MB-231 and MCF7, we did not observe any changes in the MFI dynamics between non-treated or exposed cells to diverse exosome populations. In SKBR3 cell lines, ROS intensity was decreased in the cells co-cultured with TEX but only in the Exo 2 Gy variant did this drop reach statistical significance.

One of the hallmarks of damage to DNA is the formation of DSB recognised by the presence of phosphorylated serine 139 on H2AX histone (commonly named γH2AX). In our tested BCa cell lines, we did not observe meaningful changes in the MFI levels of γH2AX after exposure to exosomal cargo derived from non- or irradiated cells. Notably, in MCF7 and SKBR3, MFI levels decreased in the cells treated with Exo 0 Gy or 2 Gy but they did not reach statistical significance.

Further, to assess the effect of exosomes on the mitochondrial conditions, we evaluated the degradation of aggregates to monomers (red to green) of JC-1 dye (5,5′,6,6′-tetrachloro-1,1′,3,3′-tetraethylbenzimi-dazolylcarbocyanine iodide), which is an indicator of depolarisation of the mitochondrial membrane (Figure 2D). In MDA-MB-231, we did not observe any significant changes referring to the decline in mitochondrial activity. Interestingly, the MCF7 cells have shown a significant drop in the red/green ratio signal in variants exposed to Exo 0 Gy or Exo 2 Gy but there was a lack of differences between them. In SKBR3, we did not observe any significant dissimilarities between the investigated variables. Another assay related to mitochondrial activity, commonly used in the determination of viability or proliferation, is 3-(4,5-dimethylthiazolyl-2)-2,5-diphenyltetrazolium bromide (the MTT assay) (Figure 2E). It could be noted that MDA-MB-231 and MCF7 cells treated with Exo 0 Gy have shown reduced levels of viability/proliferation compared to NTC or Exo 2 Gy; however, only in MCF7 was this statistically valid. In all of the tested BCa cell lines, we did not observe any changes in the percentage of viability in the Exo 2 Gy variant.

In addition, to address the possible role of exosomes derived from irradiated cell lines in the induction of radioresistance in non-irradiated cells (Figure 2F), we have tested the exposed BCa cell lines under different conditions and their survival after exposure to different doses using a clonogenic assay. We found out that in all tested BCa cell lines, there was a lack of substantial differences in response to irradiation between tested variables.

**Figure 3:**
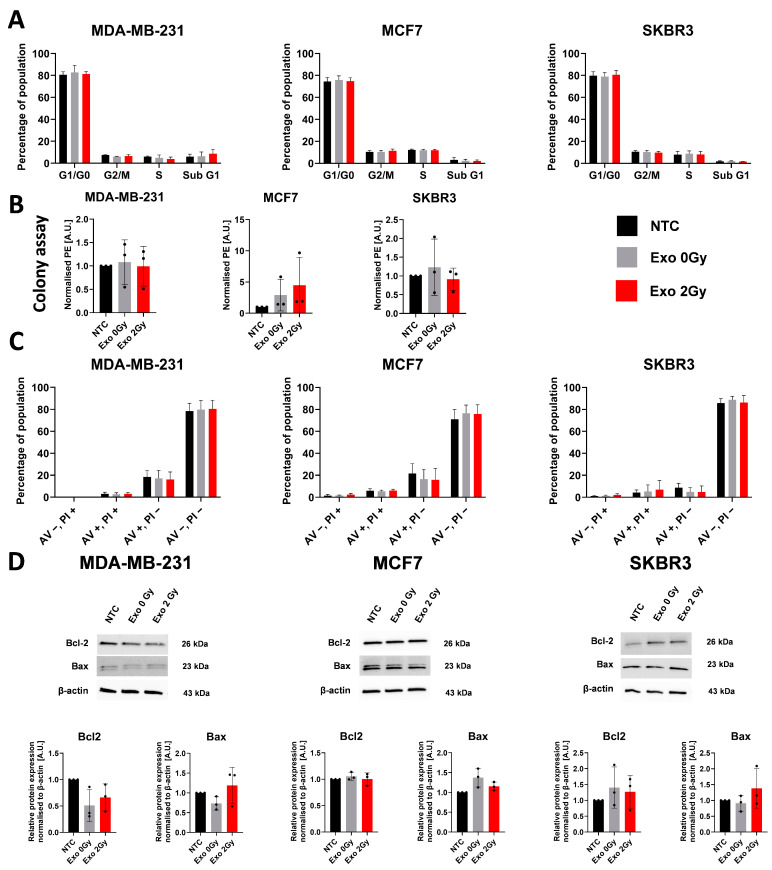
The influence of TEX derived from irradiated and non-irradiated cells onto cell cycle phases and viability of BCa. (**A**) The flow cytometric analysis of cell cycle phase distribution. The graphs represent the mean ± SD percentage of the gated phases from duplicates of three independent experiments. There was a lack of statistical differences between the tested variables. (**B**) The colony-forming assay determining the proliferation of cells co-cultured with or without exosomes. The graphs represent the mean ± SD from the triplicates (black dots) of three independent experiments. There was a lack of statistically significant differences between tested variants. (**C**) The flow cytometric analysis of apoptosis using annexin V and propidium iodide in tested cell lines. The bar charts represent the mean ± SD of the percentage of gated populations from the duplicates of three independent experiments. There was a lack of significant differences between tested variants. (**D**) The representative blots with semi-quantification of Bcl2 and Bax expression in BCa cell lines after exosome exposure, β-actin was used as a housekeeping gene. The graphs show the mean expression ± SD from the single replicate (black dot) of three independent experiments. No statistical differences were noted between tested variants.

To verify whether the cells exhibit changes in their cell cycle phase distribution, proliferation, and whether the exposure to exosomes could modify their viability, we have performed a flow cytometric analysis of the processes mentioned above, and a colony-forming assay with confirmation of the viability by estimation of the expression of B-cell CLL/lymphoma 2 (Bcl-2) and BCL2-associated X protein (Bax) (Figure 3A–D). Among all the tested cell lines and their variants, we did not notice any disturbances or changes in cell cycle phase distribution, colony formation, apoptosis, necrosis, and finally, in the expression of Bcl2 and Bax.

### 2.3. Exosomes from Non- and Irradiated Cell Lines Are Modulating the Invasive Potential of BCa Cells

**Figure 4:**
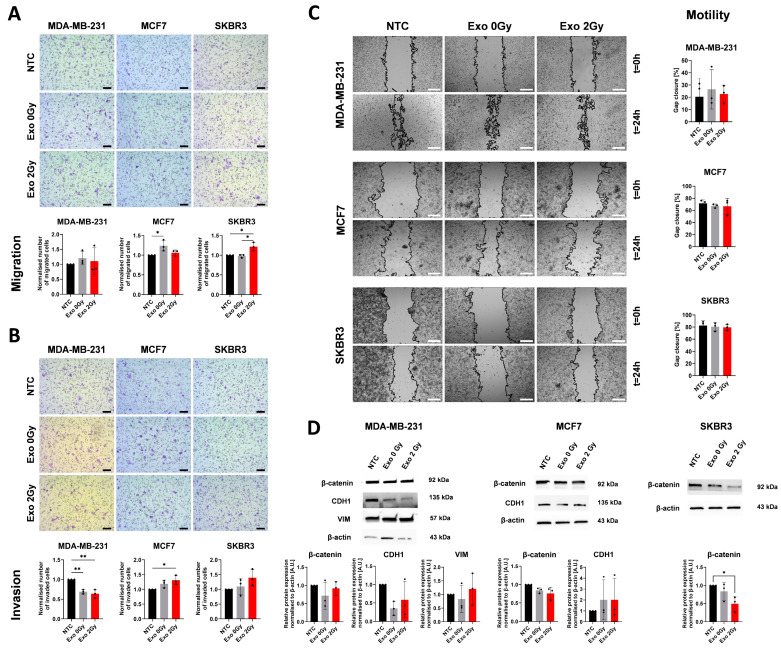
The effect of the TEX from non- or irradiated cells on migration, invasion, and motility of BCa cells. The evaluation of migration (**A**) and invasion (**B**) potential of BCa cells after exposure to the exosomes derived from non- and irradiated cells. The representative microphotographs show the migrated cells through the membrane. The black scale bar is 100 µm, and the pictures were taken under 100× magnification. The graphs represent normalised mean ± SD from three independent experiments (the black dot represents the mean from the duplicates). The statistical comparisons were calculated using ANOVA with Tukey’s post hoc test for multiple comparisons. *p* < 0.05 *; *p* < 0.01 **. PBS—control. (**C**) The analysis of motility cells using wound healing assay. The representative images present the gap closure at t = 0 and t = 24 h. The white scale bar is 100 µm. The graphs represent the mean ± SD percentage of gap closure (the black dot corresponds to the mean from technical duplicates from one independent biological experiment). (**D**) The representative blots with semi-quantification of EMT markers (β-catenin; E-cadherin—CDH1; VIM—vimentin) in BCa cell lines after exposure to exosomes from non- or irradiated cell lines, β-actin was used as a housekeeping gene. The graphs show the mean expression ± SD from the single replicate (black dot) of three independent experiments. The statistical differences were determined using ANOVA with Tukey’s post hoc test for multiple comparisons; *p* < 0.05 *.

Another crucial aspect related to malignant diseases is their ability to motile, migrate, and invade ECM. We have tested these processes using wound healing assay and migration through transwell with or without ECM. In addition, we have assessed the protein expression related to the epithelial–mesenchymal process (E-cadherin (CDH1); vimentin (VIM); and β-catenin) (Figure 4).

The migration of cells was not improved by Exo 0 Gy or 2 Gy in MDA-MB-231 cells (Figure 4A). In MCF-7, we only observed an enhanced response in cells treated with Exo 0 Gy compared to NTC. However, this change was not significant between Exo 0 Gy and Exo 2 Gy. Meanwhile, in SKBR3, the highest intensification of that process was observed in the Exo 2 Gy-treated cells. Among NTC and Exo 0 Gy, the differences were indistinguishable. The invasion of cells through ECM in MDA-MB-231 exposed to Exo 0 Gy or Exo 2 Gy was disrupted and lowered compared to NTC (Figure 4B). There was a lack of discrepancies between exosomal variants. Meanwhile, in MCF7 and SKBR3, the tendency was the opposite, where the exosomes from irradiated cells intensified their invasive properties but only in MCF7 cells were these changes statistically significant.

In addition, we performed another wound-healing assay to determine the potential for metastasis-related motility of cells (Figure 4C). In all tested cell lines and variants, there was a lack of observable changes in their motility.

To observe whether the differences mentioned above are partially related to ETM-related proteins (CDH1, VIM, and β-catenin), Western blotting was performed (Figure 4D). We did not observe VIM expression in MCF-7, CDH1, or VIM in SKBR3 cells because of their known biological background (lack of expression or mutation status). In MDA-MB-231 exposed to the distinct populations of exosomes, we observed no significant changes in examined proteins and among the tested variables. In MCF7 cells, the observable drop in β-catenin expression was noted but the results were statistically insignificant. In the case of CDH1, the expression was indistinguishable between groups. On the other hand, SKBR3 cells exhibited a significant reduction in β-catenin in Exo 2 Gy in allocation to NTC. Between the control and Exo 0 Gy, the reduction was not that high; thus, it was statistically insignificant.

### 2.4. Effect of Exosomes from Non- and Irradiated BCa Cells on Angiogenesis

**Figure 5:**
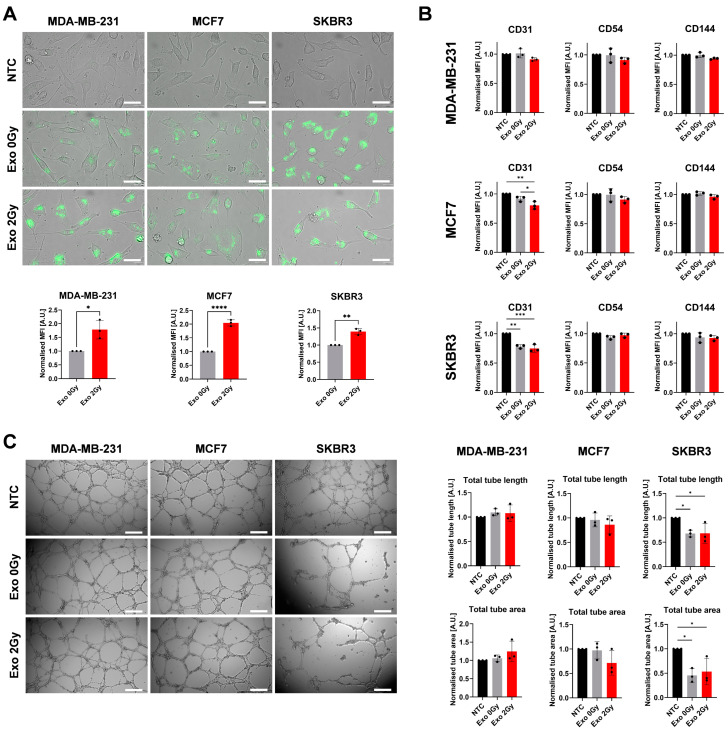
The effect of the exosomes from irradiated and non-irradiated BCa cells on angiogenesis and functions of EA.hy926 cell line. (**A**) The microscopical and flow cytometric evaluation of the labelled Exo 0 Gy or Exo 2 Gy uptake by EA.hy926 cell line after 4 h post-exposure. The representative picture of endothelial cells actively taking up PKH67-labelled exosomes. The white scale bar represents 40 µm; pictures were taken under 400× magnification. The graphs represent the normalised mean ± SD from three independent experiments (black dots represent the mean from technical duplicates) from flow cytometric MFI. The statistical significance was determined using an unpaired two-tailed Student’s *t*-test. *p* < 0.05 *; *p* < 0.01 **; *p* < 0.0001 ****. (**B**) The flow cytometric analysis of the endothelial markers CD31, CD54, and CD144. The graphs represent the normalised mean ± SD from MFI (the black dot represents the mean MFI from technical duplicates). The statistical significance was estimated using ANOVA with Tukey’s post hoc test for multiple comparisons. *p* < 0.05 *; *p* < 0.01 **; *p* < 0.001 ***. (**C**) The white scale bar represents 400 µm, and the pictures were taken under 40× magnification. The graphs represent the normalised mean ± SD (black dots represent the mean from the technical duplicates of the independent experiment) of the mean pixel area from the tube lengths or area of the formed tubes. The statistical differences between tested variants were estimated using ANOVA with Tukey’s post hoc test for multiple comparisons. *p* < 0.05 *.

One aspect related to the disease’s progression and response to radiation therapy is the modification of the vascularity and functions of the endothelial cells (EC). To determine if the exosomes from BCa from non- and irradiated cells have an impact on those processes, we have investigated their uptake and influence on a series of the proteins related to their functions (PECAM1, platelet endothelial cell adhesion molecule 1, CD31; ICAM1, intercellular adhesion molecule 1, CD54; VCAM, vascular endothelial cadherin, CD144) and tube formation assay (Figure 5).

Surprisingly, EA.hy926 cells were more prone to uptake exosomes from irradiated cells in all tested BCa cell lines (Figure 5A). Flow cytometric analysis of endothelial markers revealed that MDA-MB-231-derived exosomes had altered MFI levels, indicating a decreasing trend in cells exposed to Exo 2 Gy. However, compared to NTC or Exo 0 Gy, these changes were not statistically significant (Figure 5B). We found interesting findings in ECs exposed to exosomes from MCF-7 cells; namely, the highest drop in CD31 MFI levels was seen in the Exo 2 Gy group. No noteworthy changes were observed between Exo 0 Gy and NTC. In the case of CD54 and CD144 expression, there was a lack of statistically significant results among the variables; however, in the Exo 2 Gy variant, the MIF intensity was depleting. The exosomal cargo from non- and irradiated SKBR3 cells caused a significant drop in CD31 expression compared to NTC cells but this response was indistinguishable among groups.

Further, to determine their influence on endothelial function, we performed the tube formation assay and estimated the length and the area of the formed tube network (Figure 5C). MDA-MB-231-derived exosomes from non- and irradiated cells did not cause any distinguishable changes in the morphology and area of the developed tubes. After exposure to Exo 0 Gy and Exo 2 Gy from MCF7, EA. hy926 showed some differences from NTC, which could be noted as a decrease in the tube length and the created network’s area; however, these differences were not statistically significant. Extensive changes in ECs were observed after exposure to SKBR3-derived exosomes isolated from non- and irradiated cells. The lengths of the formed tubes and their total areas were smaller than those of NTC. Between Exo 0 Gy and Exo 2 Gy, there was a lack of significant differences.

To sum up, the data we provided suggest that the clinical dose of irradiation influences exosome secretion and the uptake of non-exposed cells. Exosomes derived from 2 Gy doses do not necessarily cause a significant effect on non-irradiated cell processes such as apoptosis and proliferation; instead, they enhance migration and invasion. More importantly, they affect the biology of endothelial cells, which could lead to the development of a proinvasive environment.

## 3. Discussion

Radiotherapy is still one of the most efficient methods of managing cancer [4]. Despite the best treatment plan, some cells could be omitted due to undetectable invasion of cells outside the tumour margins of the mass or their circulation in the system, which can cause future metastasis if they survive systemic therapy [61,62,63]. Moreover, growing evidence shows that the exposure of cells to radiation alters their biology and secretome. Its main component is exosomes, which have a major impact on recipient cells, possibly enhancing their survival [36,48,55,62,64,65,66]; on the other hand, they cause some damage, manifested as RIBE, leading to broad biological consequences in the affected cells and surrounding tissues by exosomal lipidomes, proteins, and miRNA, which shows the complex nature of exosomes [61,64,65,67]. As mentioned previously, their secretion depends on the DNA damage signalling pathway induced by irradiation [32]. In our study, we used a gamma cell—whose radioactivity source is caesium 137—generating γ-rays (energy approximately 552 kV) that in this setup are low-LET radiation, causing sparse damage to the organelles and genetic material of exposed cells, which is different from the clinically used accelerators [9,68,69]. They generate beams with higher energies and can be filtered for better control of the irradiated area, sparing healthy tissues and causing better RBE [69,70]. Exceptional examples include hadron-based therapies, which are excellent sources of high-LET radiation with significant deposition doses in the treated area (Bragg peak) [71,72]. Regarding exosomal production, recent data suggest that particle-based irradiation is a key factor in their biology and has potentially beneficial consequences for the surrounding environment by diminishing their production [55,73,74]. This is still a new area of research. However, in the case of oral cancers, the data are promising, showing that their alteration in cargo causes less immunosuppression and a lesser ability to activate signalling responses in non-exposed cells, decreasing the risk of development of radioresistance compared to the standard approach [73,74].

An increasing number of studies have shown the huge role of exosomes in cancer biology and responses to therapy [19,22,75,76]. Their cargo is sometimes acknowledged as the indicator of conditions to which cells were exposed and could provide a stable transfer of information to the recipient cell in the local or distal regions to prepare them for the incoming environmental stressor by inducing the intrinsic defences or by directly being a part of the mechanisms against the applied treatment, i.e., by directly binding to the drug or accumulating it in them to secrete outside the intracellular compartments [19]. It is a known fact that exosomal cargo could be accepted by several pathways, including docking with specific receptors on the recipient cell’s surface, endocytosis, phagocytosis, micropinocytosis, clathrin-dependent or dropping their content into the intracellular environment by direct fusion with the membrane [77]. We have determined that the irradiation has caused their increased uptake in the MCF-7 line only but the EA.hy926 cell line was observed in all Exo 2 Gy variants derived from all BC. Similar to other groups, ionising radiation was responsible for generating exosomes with an improved ability to be collected by recipient cells in a dose-dependent manner, as shown in the examples of glioblastoma, head and neck, and pancreatic cancer [45,56,57]. Only one study has revealed that their enhanced uptake could be related to the docking complex between CD29 and CD81 in irradiated mesenchymal stem cells [78]. Another reason for these properties could correspond to decreasing negative Zeta potential, which was proven to be disturbed in exosomes derived from irradiated MCF-7 cells, and its low level correlates with higher internalisation of particles [34]. This indicates that their cargo undergoes modification, which could alternate the TEX’s surface charge. One of the aspects responsible for enhanced internalisation is related to the composition of exosomal lipids. It was shown that 1 Gy of irradiation had caused an alteration within their lipidome, consequently increasing their stiffness compared to plasma lipoproteins [79]. In addition, its composition is affected by the increased amount of ceramides, which are tightly regulated by the p53-dependent pathway of exosome biogenesis known to be sensitive to the DDR caused, i.e., by irradiation [80,81,82].

It is worth mentioning that exosomal markers such as LAMP1, ALIX, and TSG101, which are connected with their biogenesis and origin, were diminished in the Exo 2 Gy variants, especially in the SKBR3 cell line. Their decreased quantity in TEX cargo and their effect on recipient cells is not well-recognised in the literature. In terms of BCa, we were able to find only one study where, similar to our study, the TSG101 levels decreased in exosomes derived from MCF-7 irradiated cells vs. those isolated from non-irradiated cells but there was a lack of explanation of this observation [48]. Similarly to our study, in two research groups with examples of head and neck cells exposed to 2 Gy and 6 Gy, respectively, TEX proteome analysis has shown some downregulation of LAMP1 and ALIX when compared to Exo 0 Gy [45]. The potential justification for these revelations could be found in the damage of the lysosomes, their involvement in membrane repair, their abruption of the endosomal sorting system, or increased heterogeneity of the exosomal cargo due to irradiation [46,67,83,84].

Recent studies have indicated that altered exosome content due to irradiation implies several consequences for non-irradiated recipient cells. In the example of the breast cancer cell line MCF-7, it was shown that their exposure to exosomes derived from 2 Gy irradiated BCa led to non-targeted effects like genomic instability, telomere shortening, and decreased telomerase activity mediated by exosomal RNA and proteins, which were persistent in these cells even after several populations doubling [44,60,65]. Furthermore, the cells exposed to irradiation could transfer signals to others through the adaptive modification of TEX cargo, resulting in the induction of radioresistance [36,44,60,65]. Their participation in determining the response to radiotherapy in human and canine BCa cells was confirmed recently [53]. Payton et al. have observed that exosomes derived from radioresistant breast cancer cells were able to induce enhanced proliferation, migration, and resistance to ionising radiation and cross-resistance to cytotoxic drugs when sensitive cells were exposed to them at a concentration of 50 µg/mL [53]. Based on those revelations, we wanted to determine whether a clinically relevant irradiation dose could also be responsible for the manifestation of some changes in non-irradiated cells. Our study did not observe such extensive manifestations regarding response to irradiation, γH2AX, proliferation, and apoptosis. One of the reasons for this outcome could be the usage of one fraction of 2 Gy irradiation, a clinically relevant dose. However, it generated significant changes in the cellular proteome, as shown in FaDu cells (head and neck cell lines) [46]. Recently, the Dong group, on the example of the MDA-MB-231 and HeLa cells exposed to 4 Gy, have shown that non-exposed cells could develop a diminished response to the applied therapy by activation of the mitogen-activated protein kinase/extracellular-signal-regulated kinase (MAPK/Erk) signalling pathway [54]. The other possible mechanism behind their increased survival and adaptation to irradiation is related to the transfer of inhibitors of apoptosis, which was confirmed in BCa exosomal cargo [55,85]. Furthermore, their enrichment and enhanced release are also induced by exposure to ionising radiation [55,66]. Another set of molecules involved in that process could be miRNA, where radioresistance can be induced by a decrease in miR-516 and miR-365 with a simultaneous increase in oncogenic miR-889 and miR-5588 in glioma exosomes [58]. We are aware that we tested only interactions between cancer cells, where the cross-talk between elements of the tumour environment could be responsible for these properties—as was shown in the example of cancer-associated fibroblasts (CAFs) in colorectal cancer—causing radioresistance through activation of the TGF-β signalling pathway or transfer miR-93-5p [86,87]. Moreover, in future studies, the effect of hypoxia should also be evaluated on the strength of induction of these mechanisms since it was reported that exosomes derived from cells cultured in hypoxic conditions also modulate the response to irradiation [88,89].

We could not determine the boosted γH2AX occurrence, a marker of DSB and typically notified as the RIBE manifestation as found in other studies [51,57,59]. A few aspects could be responsible for that fact, such as the 72 h time point in which the wave of their presence is diminished and the limitation of the flow cytometric MFI over the direct foci evaluation at single-cell resolution, which more effectively shows the possible mild changes. Furthermore, one fraction of absorbed irradiation generated a less intense effect than could be observed from a higher dose [51,57,59]. DSB induction and enhanced DDR induction were proposed as a mechanism for developing radioresistance in head and neck cancer cells in non-exposed cells via exosomal transfer [51].

We have observed some mild ROS and mitochondrial membrane potential fluctuation but in mild intensity. In the case of ROS production, the lack of observation of their increase in our study could be related to the low irradiation dose and the later time point. In contrast, in other studies, the irradiation dose was higher and was not typically used in the clinic or the individual specimen response corresponding to the origin of malignancy and their metabolic status [56,58,63]. Non-modified exosomes can induce the DDR, ROS production, and autophagy in normal breast epithelial cells to promote a pro-tumorigenic environment [90]. In glioblastomas, the induction of ROS was not distinguishable between the cells exposed to exosomes derived from irradiated cells. However, in pancreatic cancer cells, these effects were more severe, and it was shown that they could diminish superoxide dismutase 1 (SOD1) expression by transferring miR-6823-5p [56,58]. This leads to the radiosensitising effect of the cells exposed to the exosomes in a dose-dependent manner [56]. As mentioned earlier, the response of cells could be related to the metabolic status of the BCa cells, which is tightly related to mitochondrial conditions, being one of the primary sources of ROS [63,91,92,93]. The affected membrane potential in non-treated cells could be explained by the effect of uptaken exosomes themselves and caused by their influence on migration via TGF-β1, transported by TEX, as we observed promigratory and invasive potential in MCF-7 and SKBR3 [63,91,92].

Another aspect is the EMT process, which involves migration, invasion, and motility. The induction of EMT by exosomal exposure could be related to several proteins, including pleiotropic TGF-β and a plethora of miRNAs [48,94,95]. Unfortunately, in our study, we did not perform global proteomic and genomic analysis of exosomal cargo or cells exposed to their contents. Thus, we tried to present a possible mechanism behind these findings in accordance with recently published data on this subject. We have noted an interesting effect regarding MDA-MB-231 was that despite the irradiation, exosomes caused the diminished ability of cells to invade ECM. This could be related to the induction of intrinsic mechanism caused by a higher concentration of exosomes in the cell culture than it is in standard conditions, and for those revelations, a transfer of specific miRNA, i.e., miR-516, which is shown to modulate the metastasis of MCF-7 and MDA-MB-231 through modulation of β-catenin, could be one of several targets to blame [96,97]. Its presence was also confirmed in exosomes from non-irradiated glioma cells, and its content significantly decreased in a dose-dependent matter [58]. β-catenin is a crucial protein that fulfils a dualistic nature in BCa; a cytoplasmic form is combined with E-cadherin, where due to stress, its degradation is related to EMT induction [94,95,98,99]. Its downregulation in BCa causes increased invasive and migratory properties but at the cost of decreased tumorigenic potency [99]. Furthermore, its downregulation is allocated by activation of the p53-pathway, known as DDR machinery [100]. From one side, the EMT process is tightly regulated by TGF-β, which is commonly transported protein by exosomes and some WNT protein or miRNA [48,101,102]. This is in agreement with Al-Abedi et al.’s study, where MCF-7 exposed to exosomes derived from 2 Gy pre-exposed cells have shown increased invasive potential, driven by EMT-related miRNA (upregulated, miR-30a and miR9a; downregulated, miR-200b) and the involvement of TGF-β, which is enriched in exosomal compositions [48]. TGF-β-induced EMT is important in forming radioresistant BCa, creating hybrid-EMT cells and increasing the cancer stem cell expression markers [103]. Surprisingly, we did not observe the motility of cells. These discrepancies could be related to the low dose of IR as the example of head and neck cells displays that this process is dose-dependent, driven by the serine/threonine kinase Akt-signalling pathway [45]. Another explanation could be that we exposed cells directly to the exosomal cargo for the wound healing assay, whereas in migration and invasion assays, cells were used after 72 h of exposure to TEX, suggesting that the response was delayed.

Cancer cells, via exosomal cargo, can orchestrate the surrounding cells to promote their progression, development, and metastasis. One of the particular and important processes is angiogenesis [17,104,105]. To our knowledge, there is a lack of data regarding the effect of exosomes from irradiated cells on endothelial cell properties, and because of the lack of detailed information regarding exosomal composition, we tried to show the potential mechanisms of our observations. Our study showed that exosomes could affect CD31, a crucial component of blood vessel integrity and in the induction of the endothelial-to-mesenchymal transition (EndMT) [106]. Moreover, CD31 is responsible for maintaining the brain–blood barrier [107,108]. However, in this preliminary study, we did not find the explicit mechanism behind this phenomenon concerning the exosomes from irradiated cells. Interestingly, the most significant changes in the biology of endothelium cells were observed in the BCa cell lines overexpressing HER2, in which metastasis to the central nervous system (CNS) is much more frequent than in other molecular subtypes [109,110]. We suspect that this could be a consequence of a combination of several pathways, including dysregulation of the miR-126 network, long non-coding RNA (lncRNA) HOTAIR, and the involvement of TGF-β or WNT/β-catenin signalling pathway activations, which are crucial for maintenance the integrity of the vasculature and EndMT [52,106,108,111,112,113,114,115]. Moreover, the lncRNA HOTAIR have been found to correlate with the HER2 status of BCa patients and cell lines [116,117]. Its presence was confirmed in their serum-derived exosomes and correlated with the advancement of the disease and response to trastuzumab [117].

This study has several limitations related to some technical difficulties of the irradiation equipment we have used. A more translational approach could be achieved by using a clinically available accelerator. However, in that setup, we could not generate a sufficient number of plates for cell culture medium collection required for exosome isolation, whereas in the gamma cell, we could irradiate several plates simultaneously. Another angle to investigate is the high-throughput proteomic and genomic analyses of exosomal cargo, which would give us better insight into the dominant mechanisms involved in the observed changes. This could help plan additional assays that engage an overexpression system or inhibition of potential targets. Similar limitations are related to the lack of global gene expression of treated cells, which could enlighten the potentially affected signalling pathways in cancer cells after exposure to the exosomes from irradiated cells and potentially point out some molecular targets to prevent relapse of the disease and prevent the formation of radioresistant phenotypes.

In terms of future studies, the in vivo models, 3D spheroids/organoids, or patient-derived xenografts exposed to the different variants of TEX could give us insight into metastasis formation and better evaluation of tumour growth and microenvironment modifications. As an additional goal, the targeted system of delivery for inhibitors of the secretion of exosomes in irradiated cancer cells should be investigated to potentially decrease their negative impact on malignant and normal cells. Furthermore, the detailed information regarding CD31 and β-catenin downregulation targets should be studied in the exosomal cargo to decipher breast cancer cells’ novel regulatory mechanisms and invasiveness. For that purpose, high-resolution microscopy with EMT markers and changes in their phenotype should be evaluated, focusing on filopodia formation. In addition, a larger panel of HER2-positive cell lines should be evaluated regarding the formation of brain metastases, and the effect on blood barriers should be detailed to describe potential CNS tropism. Moreover, in future studies, other members of the TME, the detailed composition of the molecular cargo, long-term observations of these changes, and whether their properties change during the added fractions or the boost doses in the tumour bed could be explored. Also, interesting aspects should be evaluated in the future—the different sources of ionising radiation (i.e., proton therapy vs. photon therapy) and how the machinery of their secretion and properties are affected in non-exposed cells—to determine the novel treatment strategies overcoming the development of recurrent disease.

## 4. Materials and Methods

### 4.1. Cell Culture

The following breast cancer cell lines were used in this study (provided from the American Type Culture Collection (ATCC), Mannas, VA, USA): MDA-MB-231 (triple-negative breast cancer) (cultured in Dulbecco’s Modified Eagle Medium (DMEM), 10% Fetal Bovine Serum (FBS), and 1% Penicillin–Streptomycin (PS)), MCF7 (luminal A, estrogen receptor-positive) (cultured in DMEM, 10% FBS, 1% PS, and 0.01 mg/mL of human recombinant insulin (BIOTON, Ożarów Mazowiecki, Poland)), and SKBR3 (human epidermal growth factor receptor 2 (HER2)-positive) (cultured in DMEM mixed with F-12 nutrient mixture, 10% FBS, 1% PS, and 4 mM L-glutamine). The endothelial cell line EA.hy926 (provided from the ATCC, Mannas, VA, USA) was cultured in DMEM containing FBS 10%, non-essential amino acids (NEAA) 1×, Penicillin–Streptomycin 1×, and 4 mM L-glutamine (all of the cell culture reagents were provided by Sigma-Aldrich, St. Louis, MO, USA).

In prior experiments exposing cells to exosomes, they were washed twice with Dulbecco Phosphate Buffer (DPBS), and the fresh medium was added containing heat-inactivated FBS depleted from exosomes (Thermo Fisher Scientific Inc., Waltham, MA, USA). The final concentration of exosomes used in the study was 10 µg/mL, and co-culture lasted for 72 h. The number of cells used for each assay is reported in the Supplementary Materials, Table S1. This study used the following variants: NTC—non-treated control; Exo 0 Gy—exosomes isolated from non-irradiated BCa cells; Exo 2 Gy—exosomes isolated from irradiated BCa cells.

### 4.2. Isolation of Exosomes

The cells were seeded onto a 60 mm Petri dish and cultured up to 80% of confluence, and the following variants were collected: 1. The medium was exchanged with serum-free cell culture medium followed by twice washing with DPBS (Exo 0 Gy). 2. Prior to irradiation, culture plates were filled with DPBS (to enable the even dosage distribution) sealed with parafilm and exposed to Cs-137 (Gamma Cell^®^ 1000 Elite device, BestTheratronics Ltd., Vancouver, BC, Canada) with a dose rate of 2.5 Gy/min. The cells were exposed to the source until the absorbed dose was equal to 2 Gy (Exo 2 Gy), washed twice with DPBS, and exchanged onto serum-free cell culture medium. The medium was collected after 48 h from both variants. Exosome isolation from the cell culture medium was performed in the defined conditions as previously described [118]. Briefly, the collected and thawed media were filtered through 0.2 µm filters and reduced using 100 kDa cut-off centrifugal inserts. Then, the proper volume of concentrated medium was mixed with DPBS, administered to the ultracentrifuge test tube, equilibrated, and centrifuged for 90 min at 4 °C at 120,000× *g* (Rotor Ti 70.1, ultracentrifuge Beckman coulter L7-65; Beckman Coulter, Munich, Germany). The isolated pellet was suspended in DPBS or RIPA lysing buffer (Sigma Aldrich, St. Louis, MO, USA), depending on further usage. The concentration of the protein content was estimated using BCA assay according to the manufacturer’s instructions (Pierce™ BCA Protein Assay Kit; Thermo Scientific, Waltham, MA, USA).

### 4.3. TEM and Scanning Microscopy

The transmission and scanning electron microscopy were performed to confirm the size and morphology of isolated vesicles, as previously described with modifications [119]. The isolated exosomes were fixed using 2% paraformaldehyde, and 10 µg of the exosomes were transferred to double-sided tape adhered to the aluminium mount (Structure Probe, Inc., West Chester, PA, USA) and left overnight to dry out. The samples were covered with gold (Balzers SCD 050 sputter coater; Oerlikon Balzers, Balzers, Liechtenstein) and investigated (Scanning Electron Microscope Evo 40 Series; Carl Zeiss SMT AG, Oberkochen, Germany).

For TEM, samples were fixed using 2.5% glutaraldehyde buffer and 1% OsO4 buffer. Next, samples were dehydrated in ethanol and embedded in resin blocks. Ultrathin sections were cut with an EM UC7 ultramicrotome (Leica Microsystems, Wetzlar, Germany) onto formvar–carbon-coated copper grids and contrasted with uranyl acetate. Samples were analysed under a JEM 2100-Plus 200 kV transmission electron microscope equipped with a TVIPS TemCam–XF416 4K CMOS camera (JEOL GmbH, Freising, Germany).

### 4.4. AChE Assay

The acetylcholine esterase assay was performed to confirm the presence of the exosomes isolated from the cell culture medium as previously described [119]. The suspended 10 µg of exosomes in PBS (50 µL) was added to the 50 µL of reaction buffer containing 2.5 mM acetylcholine and 0.1 mM 5,5′-dithio-bis (2-nitrobenzoic acid) (DNTB) (all provided from Sigma-Aldrich, St. Louis, MO, USA) The mean absorbance was measured at 405 nm using the plate reader Multiskan FC (Thermofisher, San Jose, CA, USA) followed by incubation at 37 °C for 20 min. Pure PBS was used as the control.

### 4.5. MTT Assay

The viability and proliferation rate were measured using the reduction of tetrazolium salts to formazan, as previously described [120]. After 72 h exposure of BCa cell lines (seeded onto 96-well plates) to exosomes, the adequate cell culture medium (110 µL) was exchanged onto that containing 0.5 mg/mL MTT (Affymetrix, Cleveland, OH, USA) and incubated for 2 h at 37 °C. To be sure that formed crystals were dissolved using dimethyl sulfoxide (DMSO; Sigma-Aldrich, Saint Louis, MO, USA), the plate was shaken at 300 rpm for 15 min at 37 °C. The primary absorbance readout was conducted at 570 nm and the background value at 690 nm using a Multiskan FC (Thermofisher, San Jose, CA, USA).

### 4.6. Flow Cytometric Analysis

The primary procedures for cells (BCa and endothelium) were performed in accordance with previously published data [121]. Briefly, cells were dissociated using 0.25% trypsin, blocked with flow cytometry staining buffer (FCS; 2% FBS in DPBS), and washed twice with DPBS for further assays detailed below. All centrifugation steps were performed at 1500 rpm for 6 min at 4 °C and the final readout was performed on Cytoflex S (Beckman Coulter Life Sciences, Indianapolis, IN, USA) and analysed using FlowJo V10 software (FLOWJO LCC Data Analysis Software, Ashland, OR, USA).

#### 4.6.1. Annexin V and PI Staining

The viability was evaluated using flow cytometric staining—annexin V (AV) and propidium iodide (PI) (Thermofisher, San Jose, CA, USA). The prepared cells were washed in Ca^+^-containing buffer and stained for 15 min at room temperature (RT) in the dark. The readout using a flow cytometer was performed within one hour, and the percentages of the populations have been estimated and named as follows: AV−, PI− —alive; AV−, PI+—necrotic population; AV+, PI− —early apoptotic population; AV+, PI+—late apoptotic population.

#### 4.6.2. Cell Cycle Distribution Phases Analysis

The washed cells were suspended and fixed in cooled 70% ethanol by adding drops on a vortexed tube and transferred to −20 °C for at least 4 h. Then, cells were centrifuged at 3000 rpm for 10 min at RT, washed in DPBS, and stained (propidium iodide, 20 μg/mL (Cayman Chemicals, Ann Arbor, MI, USA); RNAse I, 500 μg/mL (Panreac AppliChem, Darmstadt, Germany)) at 37 °C for 30 min. The stained cells were read out immediately in flow cytometers. After the discrimination of doublets as previously described [121], the percentage of the populations in each phase was determined.

#### 4.6.3. Detection of Reactive Oxygen Species (ROS)

To evaluate the effect of exosomes on ROS production in tested BCa cell lines, they were suspended in DPBS containing 2.5 µM 2,7-dichlorofluorescein diacetate (DCFH-DA) (Sigma Aldrich, St. Louis, MO, USA) and incubated at 37 °C for 30 min. Then, cells were washed in DPBS, suspended in FCS buffer, and read immediately in a flow cytometer. The MFI levels were used for the analysis.

#### 4.6.4. Mitochondrial Membrane Potential Analysis

To evaluate the effect of exosomes on mitochondria intactness in tested BCa cell lines, they were suspended in DPBS containing 5 µM JC-1 (DCFH-DA) (Sigma Aldrich, St. Louis, MO, USA) and incubated at 37 °C for 20 min. Then, cells were washed in DPBS, suspended in FCS buffer, and read immediately in a flow cytometer; in addition, some of the suspensions were used for microscopical evaluation using an inverted-fluorescence microscope Olympus IX83 (Olympus Europa SE & Co. KG, Hamburg, Germany). The red/green ratio was determined from the MFI of PE-A (aggregates) and FITC-A (monomers) (canals were used for the analysis).

#### 4.6.5. γH2A.X Staining

Double-strand brakes (DSB) were determined using the BD Pharmingen™ Apoptosis, DNA Damage and Cell Proliferation Kit (antibody Becton Dickinson, Franklin Lakes, NJ, USA) according to the manufacturer’s instructions and recommendations. The MFI levels were used for further analysis.

#### 4.6.6. Endothelium Phenotyping

To determine the effect of exosomes on the expression of endothelial markers CD31, CD54, and CD144, the washed cells were suspended in 100 µL of FCS buffer mixed with the primary antibodies conjugated with fluorochrome (a list of antibodies and dilutions is provided in the Supplementary Materials, Table S2) and incubated for 30 min in the dark at 4 °C. The dyed cells were washed twice in FCS buffer, and the MFI levels were collected for further analysis.

### 4.7. Exosome Staining and Uptake Assay

A total of 100 µg of exosomal protein was labelled using PHK26 according to manufacturer recommendations and as previously described [25]. Further, the labelled exosomes were transferred to the mini-columns MW3000 (Invitrogen, Carlsbad, CA, USA) to decrease the excess of unbound dye. The final concentration of exosomes, 10 µg/mL, was used in uptake assays. BCa and endothelial cells were incubated for 4 h, and then pictures were taken at 400× magnification using an inverted-fluorescence contrast-phase microscope (Olympus IX83). Next, the cells were detached using Accutase, washed with PBS, and analysed using a flow cytometer. MFI levels were estimated and used for further calculations.

### 4.8. Wound Healing Assay

A wound healing assay was performed to assess the motility of cells. BCa cell lines were seeded on a 24-well plate and left overnight to attach; the fully covered well was scratched using 200 µL pipette tips. The well was washed twice with DPBS, and the fresh cell culture medium containing 10 µg/mL of exosomes was added. The pictures were taken at 0 h and 24 h post-exposure using an inverted-fluorescence microscope Olympus IX83 under 40× magnification. The gap closure was assessed using ImageJ ver. 1.54j (National Institutes of Health, Bethesda, MD, USA) plugin according to the author’s instructions and the percentage of its closure was calculated [122].

### 4.9. Migration and Invasion Assay

For the evaluation of migration and invasive potential of the BCa cells after 72 h exposure to the exosomes, they were transferred in serum-free cell culture media on previously prepared 8 µm transwell of inserts covered with liquified repellent to prevent the formation of meniscus and to help even cell distribution (Pap pen, Cancer Diagnostics Inc., Durham, CA, USA). Additionally, for the invasion assay, the inserts were covered with Matrigel (Corning Inc., Corning, NY, USA) properly diluted depending on the cell line (MDA-MB-231—1:50; MCF7 and SKBR3—1:100). As an attractant, the medium containing FBS was placed at the bottom of a 12-well plate. After overnight incubation, the cells were fixed using 100% methanol for 20 min and stained with 0.1% crystal violet solution (all purchased from Sigma-Aldrich, St. Louis, MO, USA). Then, using distilled water, the wells were washed 3 times, and the cells on the top of the insert were scraped using a cotton swab. The bottom of the insert was visualised and images were captured from at least five locations using an inverted contrast-phase microscope Axio Vert.A1 (Carl Zeiss, Oberkochen, Germany). The number of migrated/invaded cells was calculated using the Cell Counter plugin included in ImageJ ver. 1.54j.

### 4.10. Tube Formation Assay

Endothelial cells EA.hy926 were mixed with medium containing 10 µg/mL of exosomes and transferred onto a previously coated 48-well plate with a Geltrex™ LDEV-Free, Reduced-Growth-Factor Basement Membrane Matrix (Thermo Fisher Scientific Inc., Waltham, MA, USA). After 16 h, when the tubes were formed, they were documented using an inverted-fluorescence microscope (Olympus IX83) at 40× magnification. Images of the formed tubes were analysed using an AngioAnalyzer (ImageJ patch, creator Gilles Carpentier, Faculte des Sciences et Technologie, Universite Paris Est Creteil Val-de-Marne, France) [123].

### 4.11. Western Blot Analysis

To evaluate the molecular profile of the isolated exosomes, 10 µg was loaded onto the lane, and a control whole cell lysate (5 µg) was used. For the experiment regarding the incubation of BCa cells with Exo 0 Gy and Exo 2 Gy, 15 µg of protein was used per lane. The Western blotting and procedure conditions were applied as previously described [8,25]. The list of antibodies used in this study is summarised in the Supplementary Materials, Table S2. Total lane protein intensity was used for the exosomal protein cargo evaluation as the control, and for experiments regarding the exposure of BCa cells to exosomes, the protein-loading control β-actin was used.

### 4.12. Clonogenic Assay and Colony-Formation Assay

The BCa cell lines, after exposure to Exo 0 Gy and Exo 2 Gy for 72 h, were collected and irradiated in the Gamma Cell^®^ 1000 Elite device. The cells at the defined number for each dose (0 Gy, 2 Gy, 4 Gy, and 6 Gy; Supplementary Materials, Table S1) were seeded onto a 6-well plate and cultured until colonies in the control wells were composed of at least 50 cells. In addition, the non-irradiated cells (0 Gy) were used to determine their proliferation. Then, plates were fixed using 70% ethanol (for 15 min at RT) and stained with 0.2% Coomassie Blue (for 20 min at RT) (all chemicals were provided from Sigma-Aldrich, St. Louis, MO, USA). The plates were documented using the ChemiDoc Touch Imaging System (Bio-Rad Laboratories Ltd., Hercules, CA, USA), and colonies were counted using GeneTools (Syngene, Cambridge, UK). For the clonogenic assay, the survival fraction (SF) was determined by dividing irradiated cells’ plating efficiency (PE) by the PE of control cells. For the colony-formation assay, the PE between non-exposed and exosome cells was compared to those co-cultured with them.

### 4.13. Statistical Analysis

The following experiments were performed at least three times independently, processed single technical replicate or as duplicates or triplicates, as indicated in the description of the figures. The comparisons between the two groups were established using an unpaired two-tailed Student’s *t*-test; the differences between the three groups were calculated using the ANOVA test and Tukey’s post hoc test. All statistical analyses were performed using GraphPad Prism ver. 10.4.1 (627) (GraphPad Software, Boston, MA, USA).

## 5. Conclusions

The clinically relevant dose used during breast cancer treatment impacts cell biology, including the secretion and cargo of the produced exosomes, which can modulate different aspects of cancer biology of the non-exposed and endothelial cells. The findings in this study suggest their involvement in an increased migratory and invasive potential of breast cancer but not radioresistance at the used dose. Unfortunately, it still can affect the surrounding sensitive tissues, such as blood vessels, causing their decreased integrity. The heterogeneous response is related to the histological subtypes of the BCa cell lines, confirming breast cancer biology complexity. Furthermore, broad analyses related to the elements of exosomal cargo and the effects of other irradiation sources should be tested in future studies with possible targeted modifications of exosome secretion by malignant cells.

## Data Availability

The original contributions presented in the study are included in the article/Supplementary Materials, further inquiries can be directed to the corresponding author.

## Supplementary Materials

The following supporting information can be downloaded at: https://www.mdpi.com/article/10.3390/ijms26010376/s1.
