# Supplementary material for: The Effect of Ionising Radiation on the Properties of Tumour-Derived Exosomes and Their Ability to Modify the Biology of Non-Irradiated Breast Cancer Cells—An In Vitro Study
Source: Int J Mol Sci. 2025 Jan 4;26(1):376. doi: 10.3390/ijms26010376 (PMC11719956; doi:10.3390/ijms26010376)
Supplement: Supplementary file 1 [file ijms-26-00376-s001.zip › ijms-3397018-supplementary.pdf]

Table S1. The number of cells per well or test tube for experiments performed in this study.

Experiment	MDA-MB-231	MCF7	SKBR3	EA.hy926
**Uptake**	100000	100000	100000	100000
**MTT**	7000	9000	5000	-
**γH2AX staining**	200000	200000	200000	-
**ROS assay**	200000	200000	200000	-
**Cell Cycle Phases analysis**	300000	300000	300000	-
**CD31/CD54/CD144 staining**	-	-	-	200000
**Annexin V/PI assay**	200000	200000	200000	-
**JC-1 assay**	200000	200000	200000	-
**Angiogenesis**	-	-	-	45000
**Clonogenic Assay**				
0 Gy	800	1000	1200	-
2 Gy	1000	2000	2000	-
4 Gy	4000	4000	4000	-
6 Gy	6000	6000	6000	-
**Wound healing**	400000	300000	350000	-
**Invasion assay**	100000	200000	200000	-
**Migration assay**	75000	200000	200000	-

CD31 – cluster of differentiation 31 (Platelet endothelial cell adhesion molecule); CD54 – cluster of differentiation 54 (Intercellular Adhesion Molecule 1); CD144 – cluster of differentiation (VE-cadherin); γH2AX - histone gamma-H2AX; JC-1 - ; MTT - [3-(4,5-dimethylthiazol-2-yl)-2,5-diphenyl tetrazolium bromide]; PI – propidium iodide; ROS – reactive oxygen species.

Table S2. The list of antibodies used in this study.

List of antibodies	Vendor	Dilution	Host
Anti-Bcl2	Santa Cruz, Dallas, TX, USA	1:500	Mouse
Anti-BAX2	Santa Cruz, Dallas, TX, USA	1:500	Mouse
Anti-β-actin	Cell Signaling Technology, Leiden, Netherlands	1:1000	Rabbit
Anti-β-catenin	Cell Signaling Technology, Leiden, Netherlands	1:1000	Rabbit
Anti-E-cadherin	Cell Signaling Technology, Leiden, Netherlands	1:1000	Rabbit
Anti-vimentin	Cell Signaling Technology, Leiden, Netherlands	1:1000	Rabbit
Anti-TSG101	Santa Cruz, Dallas, TX, USA	1:500	Mouse
Anti-LAMP1	Santa Cruz, Dallas, TX, USA	1:500	Mouse
Anti-CD63	Santa Cruz, Dallas, TX, USA	1:250	Rabbit
Anti-CNX	Santa Cruz, Dallas, TX, USA	1:500	Mouse
Anti-Alix	Santa Cruz, Dallas, TX, USA	1:500	Mouse
Anti-TGFβ1	Cell Signaling Technology, Leiden, Netherlands	1:1000	Rabbit
Anti-VEGFA	Abcam, Cambridge, UK	1:500	Rabbit
Secondary antibodies anti-Rabbit IgG conjugated with HRP	Cell Signaling Technology, Leiden, Netherlands	1:2500	Goat

Secondary antibodies anti-Mouse IgG conjugated with HRP	Cell Signaling Technology, Leiden, Netherlands	1:2500	Horse
Anti-CD31-FITC	Beckman Coulter Immunotech, Marseille, France	1:25	Mouse
Anti-CD54-APC	BD Biosciences, Franklin Lakes, NJ, USA	1:25	Mouse
Anti-CD144-PE	Beckman Coulter Immunotech, Marseille, France	1:25	Mouse
Anti-γH2AX (ser139) - Alexa Fluor 647	BD Biosciences, Franklin Lakes, NJ, USA	1:25	Mouse
APC isotype control	BD Biosciences, Franklin Lakes, NJ, USA	1:25	Mouse
PE isotype control	BD Biosciences, Franklin Lakes, NJ, USA	1:25	Mouse
FITC isotype control	BD Biosciences, Franklin Lakes, NJ, USA	1:25	Mouse
Alexa Fluor 647 isotype control	BD Biosciences, Franklin Lakes, NJ, USA	1:25	Mouse

APC - Allophycocyanin; BAX - BCL2-associated X protein; BCL2 - B-cell CLL/lymphoma 2; CD31 – cluster of differentiation 31 (Platelet endothelial cell adhesion molecule); CD54 – cluster of differentiation 54 (Intercellular Adhesion Molecule 1); CD63 cluster of differentiation (tetraspanin); CD144 – cluster of differentiation (VE-cadherin); CNX - calnexin; FITC – fluorescein isothiocyanate; γH2AX - histone gamma-H2AX; HRP - horseradish peroxidase; LAMP1 - lysosome-associated membrane glycoprotein 1; PE - phycoerythrin ; TGF-β1 - transforming growth factor beta-1; TSG101 - tumour susceptibility gene 101 ; VEGFA - vascular endothelial growth factor A.
